# Bone Healing of Critical-Sized Femoral Defects in Rats Treated with Erythropoietin Alone or in Combination with Xenograft

**DOI:** 10.3390/vetsci10030196

**Published:** 2023-03-05

**Authors:** Radina Vasileva, Tzvetan Chaprazov

**Affiliations:** Department of Veterinary Surgery, Faculty of Veterinary Medicine, Trakia University, Stara Zagora 6000, Bulgaria

**Keywords:** erythropoietin, xenograft, bone regeneration, femur, rat

## Abstract

**Simple Summary:**

The treatment of large bone defects caused by fractures is still a challenge in orthopaedics. In this study, the potential of erythropoietin to promote bone regeneration was investigated in an experimental rat model. Bone healing was monitored through objective radiological, osteodensitometric and histological methods. The assessment suggests that erythropoietin, applied locally on a collagen scaffold alone or in combination with a bone substitute, is capable of inducing bone healing in femoral defects.

**Abstract:**

Critical-size bone defect models are the standard in studies of the osteogenic potential of biomaterials. The present investigation aimed to evaluate the ability of recombinant human erythropoietin (EPO) to induce trabecular bone healing either alone or combined with a xenograft in a rat femoral critical-size defect model. Five-mm bone defects were created in the femoral diaphysis of fifty-six skeletally mature male Wistar albino rats. The animals were divided into six groups: one control group and five experimental groups. The defects in the control group were left empty, whereas an absorbable collagen cone soaked either with saline or erythropoietin (alone or in combination with xenograft) was placed in locally treated groups. The systemic treatment group received EPO subcutaneously. Bone formation was objectively evaluated through radiography, osteodensitometry and histological examination on post-operative days 30 and 90. The results demonstrate that EPO, locally applied on a collagen scaffold, was capable of inducing bone healing, whereas the single systemically administered high EPO dose had only an insignificant effect on bone formation. The combination of EPO with a bone substitute under the form of cancellous granules resulted in more rapid integration between the xenograft and host bone.

## 1. Introduction

Critical-size bone defects are the smallest bone injuries that are not able to heal spontaneously [1]. In human orthopaedics, a critical defect’s deficiency length must exceed 2 cm, or there must be a loss of more than 50% of bone diameter [2,3]. In preclinical studies involving animals, defects should be not able to heal spontaneously throughout the duration of the experiment in order for them to be defined as critical-size [4].

The therapeutic approach to the treatment of such extensive defects requires the use of some acknowledged bone reconstruction methods as distraction osteogenesis or bone transplantation, including autogenous bone grafts, bone marrow aspirate, allografts, bone substitutes and growth factors [5,6,7,8]. The clinical results, however, are not always satisfactory, necessitating the search for new osteogenic factors or molecules for clinical orthopaedic application.

Erythropoietin (EPO) is a cytokine, possessing a number of additional functions apart from its haematopoietic effect [9,10]. Its in vitro effect on bone tissue comprises the stimulation of mesenchymal stem cells transformation into osteoblasts [11], and its in vivo effects include the proliferation of osteoblasts, enhanced bone formation, increased callus mineralisation rate, increased bone density and bone mechanical strength [12,13,14].

The information on the combined utilization of EPO and xenografts is scarce [15]. Recent studies showed that EPO reduced the time for integration of the bone substitute through improvement of vascularization [16,17]. This is probably due to its angiogenic potential, which is similar to that of the vascular endothelial growth factor [18,19].

The effects of local EPO application on the healing of long tubular bones in animals were investigated with radial, femoral and tibial models. The osteotomy of the radius or ulna does not require fixation of the defect due to the lack of supination and the presence of radioulnar interosseous ligament, contributing to good bone stability [20]. The main disadvantage of the model from a clinical point of view is that the load on both bones is relatively small [21]. That is why the optimum experimental design in studies on regeneration of weight-bearing bones consists of the creation of defects in the femur with subsequent fixation [22]. In rats, both femoral diaphysis and metaphysis are appropriate, yet the former is preferred due to less difficult bone fixation [23].

Conventional radiography [24,25], computed tomography [26,27] and dual-energy X-ray absorptiometry (DEXA) [28,29] are methods for bone healing monitoring. According to available data in the literature, DEXA may be used for the evaluation of callus mineralization and is more sensitive than conventional radiography for detection of bone formation disorders [30].

The present investigation aimed to evaluate the ability of erythropoietin to induce bone healing of rat femoral critical-size defects, either alone or combined with a bone substitute, through objective radiological, osteodensitometric and histological evaluation of bone formation.

## 2. Materials and Methods

### 2.1. Experimental Animals

Fifty-six skeletally mature male Wistar albino rats with an average weight of 270 ± 25 g were used. Throughout the experiments, the rats were housed in the Biobase of the Faculty of Veterinary Medicine, Trakia University in laboratory polypropylene rat cages with grills; the cages were 427 mm × 287 mm × 198 mm, according to conditions listed in Appendix 4 of Ordinance 20 of 1.11.2012. The experiments were approved by the Ethics Committee of the Bulgarian Food Safety Agency and Ordinance 25 of 10.06.2005 for the protection and welfare of experimental animals. The permit is entered in the BFSA register under reference No. 276.

### 2.2. Materials

#### 2.2.1. Erythropoietin

In this study, 2000 IU Binocrit injection solution containing 2000 IU/mL epoetin alpha, equivalent to 16.8 μg/mL (Sandoz GmbH, Kundl, Austria), was used. It was produced by using recombinant DNA technology in the Chinese Hamster Ovary cell line expression system.

#### 2.2.2. Collagen Matrix

Collacone^®^ (Botiss biomaterials GmbH, Berlin, Germany) is a collagen cone on the basis of porcine collagen, which is entirely absorbed in the body for 2–4 weeks.

#### 2.2.3. Bone Substitute

The bone substitute Bio-Gen^®^ (BiOTECK, Arcugnano, Italy) consists of spongy granules of equine origin. The time for its remodelling ranges from 4 to 6 months.

#### 2.2.4. Metal Implants

The osteosynthesis of bone defects was performed with mini bone plates (30 mm length; 4 mm width) and four cortical screws with a diameter of 1.5 mm and lengths of 6 or 8 mm (Biortho-Vet-Eur, Jiangsu, China).

### 2.3. Anaesthesiological and Surgical Protocol

**Figure 1:**
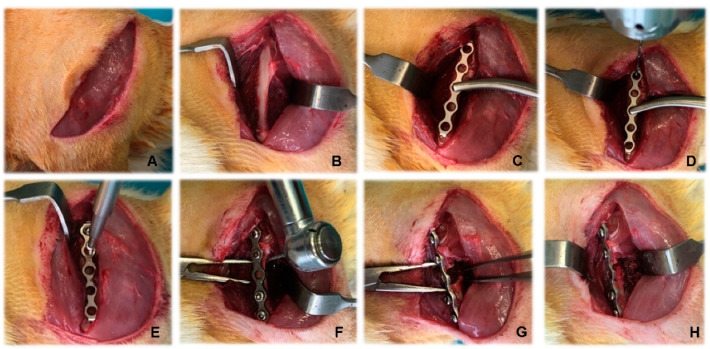
Stages of creation of rat femoral segmental critical-size defects. (**A**) Skin incision; (**B**) approach to the femoral shaft; (**C**) placement of plate; (**D**) drilling of holes for the screws; (**E**) placement of screws; (**F**) creation of the critical-size defect; (**G**) exposure of the defect; (**H**) placement of biomaterials.

The animals were anaesthetized via intramuscular injection of 80 mg/kg 10% ketamine hydrochloride (Anaket^®^, Richter Pharma AG, Wels, Austria) and 10 mg/kg 2% xylazine hydrochloride (Xylazin^®^, Alfazan, Woerden, The Netherlands). Before the surgery, rats were injected with 10 mg/kg of the antibiotic enrofloxacin (Baytril^®^, Bayer, Leverkusen, Germany) and 0.08 mg/kg of the analgesic buprenorphine (Bupaque^®^, Richter Pharma AG, Wels, Austria). The entire left pelvic limb was shaved, and the skin was disinfected with 10% povidone–iodine (Braunol, B. Braun Melsungen AG, Melsungen, Germany). After a routine lateral approach to the femoral shaft [31] and bone fixation, a 5 mm critical-size segmental defect was made using a neuro bur under continuous irrigation with 0.9% saline solution. The main stages of the surgery are illustrated in Figure 1. During the first three postoperative days, the rats were administered antibiotics orally with their drinking water.

**Figure 2:**
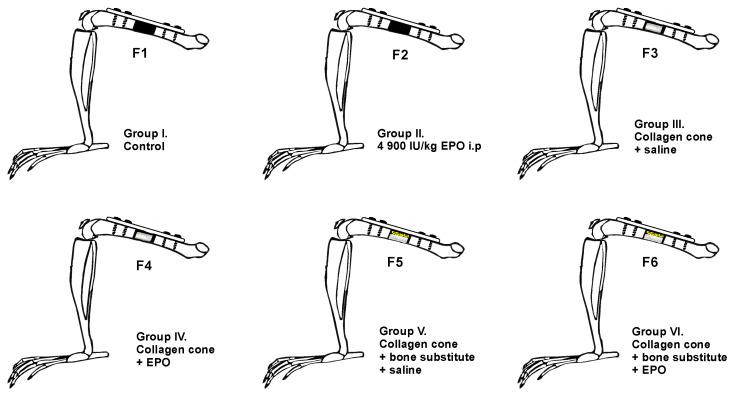
Experimental design: schematic presentation of groups and defects.

Laboratory animals were divided into six groups: one control group and five experimental groups (Figure 2). After the creation of the defect, the following biomaterials were placed at the ostectomy site:

- Group F1 (*n* = 10): the defect was left empty.
- Group F2 (*n* = 10): the defect was left empty, and the rats received a single intraperitoneal EPO injection at a dose of 4900 IU/kg.
- Group F3 (*n* = 8): collagen cone soaked with physiological saline.
- Group F4 (*n* = 10): collagen cone soaked with 49 IU EPO.
- Group F5 (*n* = 8): bone substitute combined with collagen cone soaked with physiological saline.
- Group F6 (*n* = 10): bone substitute combined with collagen cone soaked with 49 IU EPO.

### 2.4. Radiography

Craniocaudal radiographs of the femoral region were taken immediately after the operation and on post-operative days 30 and 90 in ketamine/xylazine-sedated animals by means of the PHILIPS SUPER 50 CP-D digital X-ray system (Hamburg, Germany) with exposure data 45 kV, 8 mAs.

Relative bone density (RBD) was assessed on digital radiographs by means of the Image J software (ImageJ Analysis System, National Institutes of Health, Bethesda, USA) and expressed as a ratio of the mean gray value of the defect and the mean gray value of the surrounding bone tissue. One range of interest entirely corresponding to the defect size was created, whereas the “Freehand Selection” tool of the software was used to create another range of interest for the surrounding bone.

The healing of bone defects was evaluated and scored on radiographs using the radiological criteria proposed by Oryan et al. (2015) [32] with modifications. Individual scores (from 9 to 37) for each experimental animal were obtained as a sum of all criteria (Table S1).

### 2.5. Bone Densitometry

The femoral bone mineral density of rats from experimental groups (F4–F6) was measured on the 90th post-operative day after the removal of metal implants through dual-energy X-ray absorptiometry (DEXA). Scans were performed with an OsteoSys SMART FAN-BEAM densitometer (OsteoSys, Seoul, Korea). Bone mineral density data were generated with the Excellus software (OsteoSys, Seoul, Korea) and are presented as g/cm^2^.

### 2.6. Histological Examinations

Half of the animals from each experimental group were euthanised on the 30th post-operative day (*n* = 28), and the other half were euthanised on the 90th day (*n* = 28). Euthanasia was carried out via of isoflurane overdose by inhalation (AErrane 100% Liquid Inhalation Vapour, Baxter, Norfolk, UK) [33]. Femur specimens were fixed in 10% neutral formalin, decalcified and embedded in paraffin blocks for subsequent staining with haematoxylin-eosin (H&E) and Masson-Goldner trichrome (MT).

Histological preparations were observed on a Leika DM1000 binocular light digital microscope (Leica Microsystems, Milton Keynes, UK), and findings were microphotographed at magnifications ×40, ×100 and ×200 by using a LEICA DFC 290 digital camera (Leica Microsystems, Miton Keynes, UK). The presence of newly formed bone and bone substitute remnants was observed.

### 2.7. Statistical Analysis

The results for relative bone density and healing scores are presented as medians (minimum–maximum). Due to the different sample sizes in each group, the differences between individual groups at a given period were evaluated by using the Mann–Whitney test, and the changes with time within each group were evaluated by using the Kruskal–Wallis test with Dunn’s post hoc test. All analyses were performed with statistical software MedCalc v.15.8 (MedCalc Software Ltd., Ostend, Belgium).

## 3. Results

### 3.1. Clinical Examinations

During the post-operative period, the rats were in good general condition with preserved appetites. Moderate swelling was present in the femoral region of the operated limb, which resolved within 3–5 days. During the first post-operative day, the rats bore weight on the operated limb with low-grade lameness, which disappeared after the 3rd post-operative day.

### 3.2. Radiography

**Figure 3:**
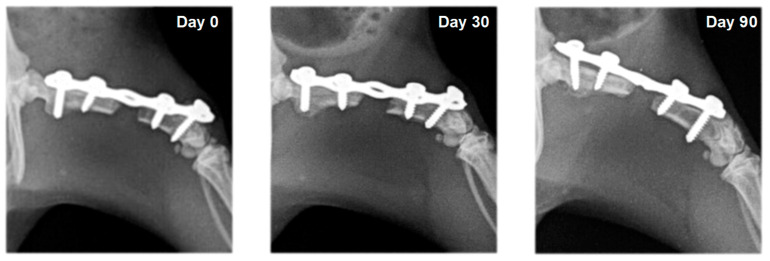
Serial craniocaudal radiographs of rat femurs from experimental group F1 on post-operative days 0, 30 and 90.

**Figure 4:**
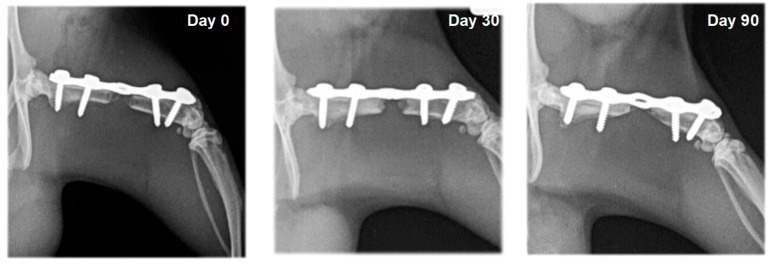
Serial craniocaudal radiographs of rat femurs from experimental group F2 on post-operative days 0, 30 and 90.

In rats from the control group (F1) and the group with systemic EPO application (F2), bone formation signs were not established. In the different study periods, osteotomy lines and the created segmental defects were clearly visible (Figure 3 and Figure 4).

The radiological bone healing scores, according to Oryan et al. (2015), in the different groups are presented in Table 1. In rats from the group with local EPO application (F4), bone formation was induced around the osteotomy margins, with statistically significant differences on post-operative days 30 and 90 vs. day zero. Compared to group F3 (collagen cone soaked with physiological saline), the difference of healing scores in group F4 by the 90th day was statistically significant (*p* < 0.05), which was associated with reduced bone defect size and the formation of bone bridges (Table 1).

**Figure 5:**
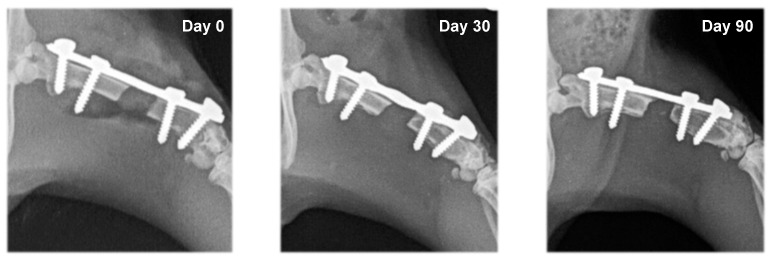
Serial craniocaudal radiographs of rat femurs from experimental group F3 on post-operative days 0, 30 and 90.

**Figure 6:**
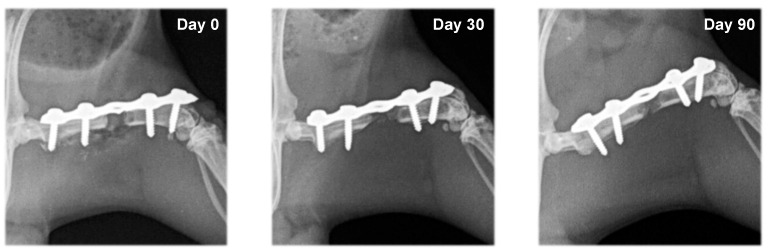
Serial craniocaudal radiographs of rat femurs from experimental group F4 on post-operative days 0, 30 and 90.

On radiographs, newly formed bone appears as a non-homogenous increased radiopacity (Figure 5 and Figure 6).

**Figure 7:**
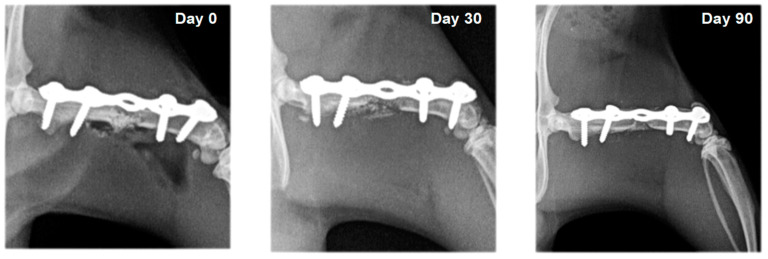
Serial craniocaudal radiographs of rat femurs from experimental group F5 on post-operative days 0, 30 and 90.

**Figure 8:**
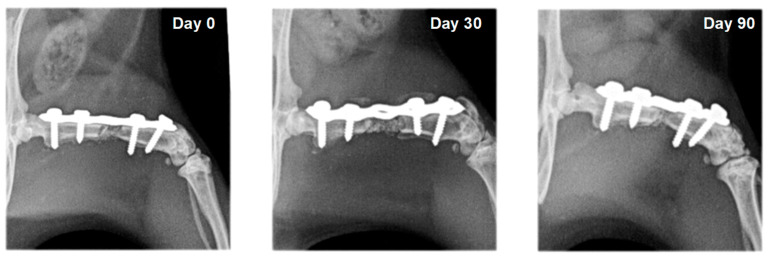
Serial craniocaudal radiographs of rat femurs from experimental group F6 on post-operative days 0, 30 and 90.

In rats whose defects were filled with bone substitute, the xenograft was visualized with strong heterogeneous radiopacity and was radiologically visible until the end of the experiments. Bone integration between the xenograft and the host bone was not observed in rats from group F5, which were treated with bone substitute and saline. Bone formation was evident in animals from group F6, whose defects were filled with bone substitute and erythropoietin (Figure 7 and Figure 8). A statistically significant difference (*p* < 0.05) between the healing scores of these two groups was observed on the 30th post-operative day (Table 1).

The relative bone density (RBD) values changed in all groups throughout the experiments (Table 2). The analysis of the results demonstrates statistically significant differences in the average RBD of groups F1–F4 by the 30th day and by the 90th day vs. day zero (*p* < 0.001). Values on the 90th day were substantially higher than those measured on post-operative day 30 (*p* < 0.001 for groups F1, F2 and F4; *p* < 0.01 for group F3). In groups F5 and F6, RBD also tended to increase, yet the values on post-operative day 90 did not differ significantly from the preceding survey periods.

Average RBD values of control rats (F1) and those treated i.p. with EPO (F2) differed significantly only on the 30th post-operative day (*p* < 0.01). The RBD values in group F3 (collagen cone soaked with saline) and group F4 (collagen cone soaked with EPO) were statistically different by the 90th day (*p* < 0.05), as were the RBD values of groups F5 (bone substitute with collagen cone soaked with saline) and F6 (bone substitute with collagen cone soaked with EPO) (*p* = 0.05).

### 3.3. Bone Densitometry

The results from the DEXA scans show that the bone densities of left femoral bones that underwent bone regeneration were higher than those of right healthy contralateral bones (Table 3). The differences among the different experimental groups were not statistically significant. The average bone mineral densities of the left and right pelvic limbs differed considerably (*p* < 0.05) in rats from group F6, which were treated with bone substitute combined with a collagen cone soaked with erythropoietin.

### 3.4. Histology

#### 3.4.1. Microscopic Histological Pattern on Post-Operative Day 30

**Figure 9:**
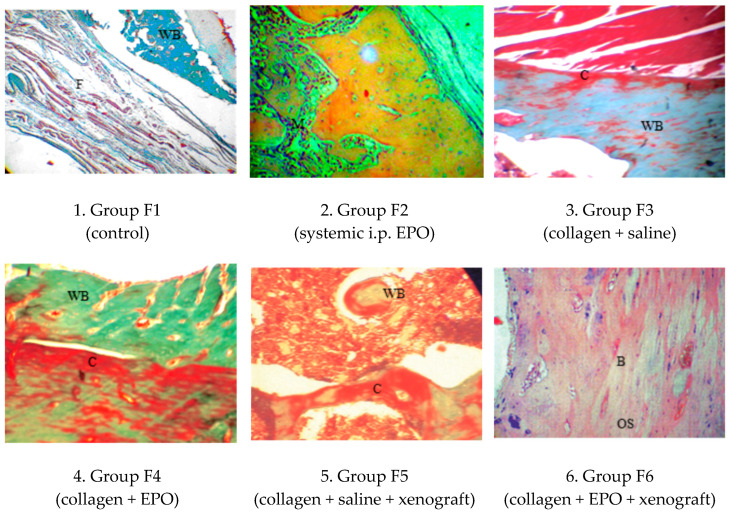
Histological patterns of rat femur segmental defects in different study groups on day 30. (1) Muscle and collagen fibres (F) surrounding a scarce woven bone (WB), MT × 100; (2) mesenchyme (M) is directed from the periphery towards the centre of the defect, H&E × 125; (3) newly formed woven bone (WB) and neovascularization, MT × 125; (4) contact between the collagen cone (C) and the newly formed woven bone (WB), MT × 125; (5) collagen cone (C) and woven bone (WB), undergoing transformation into lamellar bone, MT × 125; (6) the newly formed bone matrix (B) with osteocytes (OS) residing in lacunae, H&E × 125.

The cross-sectional surface of purple-red-stained microelements of the collagen scaffold were well delineated, as they were most commonly in contact with the bioptate’s cartilaginous fragments. In most histological cross-sections, cartilaginous fragments exhibited morphological signs of transformation into lamellar bone at various stages (Figure 9).

#### 3.4.2. Microscopic Histological Pattern on Post-Operative Day 90

**Figure 10:**
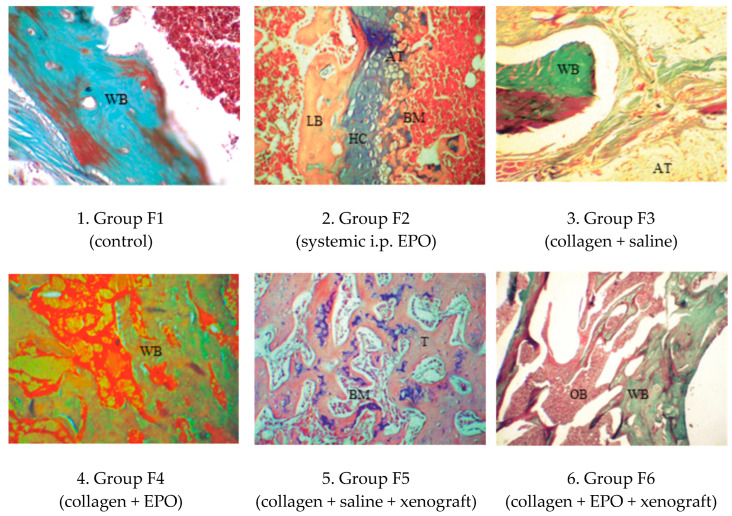
Histological patterns of rat femur segmental defects in different study groups on day 90. (1) Hyaline and fibrous cartilage transformation into lamellar bone with visible bone marrow spaces, MT × 125; (2) the bony and cartilaginous parts (HC) are adjacent to differentiating red bone marrow (BM) and white adipose tissue, H&E × 125; (3) woven bone (WB), surrounded by amorphous substance, cells and adipose tissue (AT), MT × 100; (4) woven bone (WB) undergoing transformation into lamellar bone, MT × 100; (5) trabeculae and bone marrow, H&E × 120; (6) all cavities demonstrate transformation of cartilage into bone tissue, MT × 120.

From connective tissue lining towards bioptate cores, all examined sections demonstrated active processes of transformation of cartilage into bone, the initial differentiation of woven into lamellar bone and the initial formation of medullary cavity network and bone marrow (Figure 10).

## 4. Discussion

Models developed for assessment of the effects of EPO application on long tubular bone healing involve closed fracture, osteotomy or ostectomy with the creation of segmental bone defects [34]. In this study, the potential of erythropoietin to induce new bone formation was evaluated on the basis of 5 mm critical bone defects in the femoral diaphysis of rats.

Native radiography is a primary technique for monitoring fracture healing and bone defect regeneration [35]. The performed serial radiographic examinations revealed various tendencies in the different experimental groups. Bone healing signs were not found on the radiographs of the control group of rats, confirming the fact that the 5 mm femoral diaphyseal defect in this animal species was of critical size. The increased values of relative bone density corresponded to osteosclerosis occurring on the margins of proximal and distal fragments, which is a main sign of a narrowing of the medullary cavity. The findings were the same in rats from group F2, which were treated intraperitoneally with EPO. Conversely to these observations, Holstein et al. (2007) reported greater callus volume in mice treated intraperitoneally with 5000 IU/kg EPO for six consecutive days compared to untreated controls [13]. Several years later, they performed another experiment with murine femurs, but EPO was applied at a tenfold lower dose (500 IU/kg) and over a rather prolonged period (2–10 weeks). This time, the EPO-treated groups demonstrated considerably faster bridging with new bone tissue formation and greater callus volume than the controls [36]. The discrepancy between these results and the present findings may be attributed to the different experimental designs: the first model of Holstein et al. used a closed fracture, and the second used a segmental defect, whereas the present study used a critical-size bone defect, presuming a different duration of regeneration.

The placement of collagen cone soaked with saline at the osteotomy site turned out to be insufficient for bone healing, yet it influenced relative bone density, allowing attachment of cells and new tissue formation [37].

The radiographs of rats from group F4 revealed signs of callus formation as early as the 30th post-operative day. The callus of both bone fragments grew gradually towards the centre of the defect, and bone union occurred by the 90th day. This is supported by the statistically significant changes in relative bone density. Our data are comparable with those of Omlor et al. (2016), who observed signs of bone formation along the radial ulnar surface after local application of 4900 IU/kg EPO on a gelatin sponge with or without medullary cavity formation. In addition, a greater bone callus volume was reported [38].

Previous studies confirmed bone healing enhancement with xenografts [39,40]. Unfortunately, this study showed that their independent application was not satisfactory. Spongy granules were radiologically visible throughout the monitoring period, were responsible for the higher relative bone density values and underwent remodelling, but they adhered to the host bone to an insufficient extent, which delayed healing [41,42]. Karalashvili et al. (2017) used a bovine bone graft for zygomatic bone reconstruction and reported that it retained its shape without resorption and bone integration [43]. Substantially better results were achieved by Kharkova et al. (2019) after combining a tricalcium phosphate scaffold with EPO. The researchers reported that this complex was promising for healing radial bone defects in rats, which supports the better effect from the xenograft and EPO co-administration observed in the present study [16].

Dual-energy X-ray absorptiometry (DEXA) was used for the quantitation of bone mineral density (BMD), an important parameter for the prediction of fracture risk and for monitoring bone healing. BMD’s diagnostic precision is good and correlates with the biomechanical properties of bone [44]. According to Markel et al. (1995) DEXA may be used for the evaluation of ostectomy healing and the prediction of complications [28]. The observed higher BMD values of operated limbs vs. intact contralateral limbs confirmed the study results of Li et al. (2005): significantly increased BMD at the fracture line for operated limbs, showing a tendency towards healing. At the same time, the authors reported that bone callus BMD was 29.5%, 48.3%, 85.3% and 105.2% higher than contralateral limb BMD values, as measured on post-fracture weeks 2, 4, 6 and 8, respectively. With time, bone mineral density correlated with histomorphological features at the healing fracture site [30].

The histological patterns of rat femoral bone specimens demonstrated that the regeneration of the critical-size femoral defect occurred via endochondral ossification. This is in line with earlier experiments involving radial critical-size bone defects in rats [16]. Likewise, 30 days after the surgery, the gap was filled with cartilaginous callus showing morphological signs of transformation into lamellar bone but to different extents, depending on the experimental group. The process was the most obvious in the group in which EPO was applied locally on a collagen scaffold either with or without a xenograft. Garcia et al. (2011) also reported improved bone healing after femoral ostectomy, with a fracture gap of 0.25 mm [14]. Compared to the present findings, the authors described greater bone volume and fewer connective tissue at the site of the osteotomy or fracture gap. In contrast to our results, the proportion of cartilaginous callus was smaller, which is probably due to the fact that the used model did not involve a critical-size bone defect.

The quantitation of blood vessels on histological sections performed by Omlor et al. (2016) shows that EPO-induced vascularization is another important mechanism for bone healing enhancement [38]. Similar to our findings, they also observed substantially enhanced blood vessel formation 12 weeks after either local or systemic application of a single EPO dose of 4900 IU/kg.

The findings of this study demonstrate the beneficial effect of erythropoietin applied locally on a collagen scaffold to manage critical-size long-bone defects, either alone or in combination with a xenograft. However, other animal models and longer follow-up periods are needed to investigate the credibility of the obtained results regarding the clinical implementation of the presented outcomes.

## 5. Conclusions

The integral assessment of the regeneration potential of erythropoietin in a rat femoral critical-size defect model allows us to conclude the following:

1. Erythropoietin, applied locally on a collagen scaffold, is capable of inducing bone healing, whereas a single high dose applied intraperitoneally has only an insignificant effect on bone formation.
2. Erythropoietin results in a more rapid integration of spongy xenograft granules to the host bone.
3. Histologically, the regeneration of rat femoral critical-size defects occurred via endochondral ossification.
4. Osteodensitometry, a non-invasive technique of quantifying bone mineral density, may be used to assess the strength of the newly formed bone callus.

## Figures and Tables

**Table 1:** Radiological bone healing scores of critical-size segmental femoral defects in rats from the different experimental groups. Data are presented as medians (minimum–maximum).

Group	Day 0	Day 30	Day 90
F1	*n* = 10	*n* = 10	*n* = 5
	9 (9–9)	11 (11–11) ^a***^	11 (11–11) ^a**^
F2	*n* = 10	*n* = 10	*n* = 5
	9 (9–9)	11 (11–11) ^a***^	11 (11–11) ^a***^
F3	*n* = 8	*n* = 8	*n* = 4
	9 (9–9)	15 (11–16) ^a**^	16.5 (14–19) ^a** b*^
F4	*n* = 10	*n* = 10	*n* = 5
	9 (9–9)	16.5 (11–25) ^a**^	22 (21–32) ^a***^
F5	*n* = 8	*n* = 8	*n* = 4
	11 (11–11)	25.5 (22–28) ^a*** c*^	24.5 (21–30) ^a**^
F6	*n* = 10	*n* = 10	*n* = 5
	11 (11–11)	28 (25–34) ^a***^	32 (25–34) ^a**^

^a**^ *p* < 0.01; ^a***^ *p* < 0.001 vs. day 0 within each group; ^b*^ *p* < 0.05 between experimental groups F3 and F4 at each period; ^c*^ *p* < 0.05 between experimental groups F5 and F6 at each period.

**Table 2:** Relative bone density of critical-size segmental femoral defects in rats from the different experimental groups. Data are presented as medians (minimum–maximum).

Group	Day 0	Day 30	Day 90
F1	*n* = 10	*n* = 10	*n* = 5
	0.60 (0.47–0.63)	0.66 (0.64–0.71) ^a***^	0.75 (0.73–0.77) ^a*** b***^
F2	*n* = 10	*n* = 10	*n* = 5
	0.52 (0.43–0.63)	0.71 (0.69–0.73) ^a***^	0.76 (0.73–0.78) ^a*** b***^
F3	*n* = 8	*n* = 8	*n* = 4
	0.66 (0.54–0.68)	0.70 (0.65–0.74) ^a***^	0.75 (0.73–0.77) ^a*** b**^
F4	*n* = 10	*n* = 10	*n* = 5
	0.65 (0.60–0.69)	0.72 (0.68–0.76) ^a***^	0.85 (0.77–0.86) ^a*** b***^
F5	*n* = 8	*n* = 8	*n* = 4
	0.86 (0.74–0.88)	0.87 (0.84–0.94)	0.92 (0.87–0.96)
F6	*n* = 10	*n* = 10	*n* = 5
	0.87 (0.75–0.90)	0.92 (0.84–0.95)	0.98 (0.90–0.99)

^a***^ *p* < 0.001 vs. day 0 within each group; ^b**^ *p* < 0.01; ^b***^ *p* < 0.0001 vs. day 30 within each group.

**Table 3:** Bone mineral density (g/cm^2^) of rat femurs from experimental groups F4, F5 and F6 at post-operative day 90. Data are presented as medians (minimum–maximum).

	Group F4 *n* = 5	Group F5 *n* = 4	Group F6 *n* = 5
Right femur	0.303 (0.217–0.396)	0.318 (0.240–0.384)	0.320 * (0.266–0.396)
Left femur	0.378 (0.335–0.408)	0.400 (0.311–0.420)	0.430 (0.380–0.513)

* *p* < 0.05 between right and left femurs for a given group.

## Data Availability

The data presented in this study are available upon request from the corresponding author.

## Supplementary Materials

The following supporting information can be downloaded at: https://www.mdpi.com/article/10.3390/vetsci10030196/s1, Table S1: Scoring system for evaluation of the fracture healing of bone defects on radiographs.
