# Supplementary material for: Bone Healing of Critical-Sized Femoral Defects in Rats Treated with Erythropoietin Alone or in Combination with Xenograft
Source: Vet Sci. 2023 Mar 5;10(3):196. doi: 10.3390/vetsci10030196 (PMC10056540; doi:10.3390/vetsci10030196)
Supplement: Supplementary file 1 [file vetsci-10-00196-s001.zip › vetsci-2239223-supplementary.pdf]

Table S1. Scoring system for evaluation of the fracture healing of bone de-fects on radiographs.

Criterion	Score
1. Bone formation	
No evidence of bone formation	1
Bone formation occupying 25% of defect	2
Bone formation occupying 50% of defect	3
Bone formation occupying 75% of defect	4
2. Total radiographic union	
Nonunion	1
Possible union	2
Radiographic union	3
3. Proximal osteotomy union	
Nonunion	1
Mild bridge (<50%)	2
Moderate bridge (>50%)	3
Union	4
4. Distal osteotomy union	
Nonunion	1
Mild bridge (<50%)	2
Moderate bridge (>50%)	3
Union	4
5. Bridging	
No bridging	1
<50% bridged	2
>50% bridged	3
Fully bridged	4
Normal cortical morphology	5
6. Fracture lines	
Fracture line from 1.0 to 5.0 mm without bone proliferation	1
Fracture line from 1.0 to 5.0 mm, with bone proliferation, without bridging callus	2
Fracture line from 1.0 to 5.0 mm, with bone proliferation and bridging callus	3
Fracture line <1.0 mm, without bone proliferation	4
Fracture line <1.0 mm, with bone proliferation, without bridging callus	5
Fracture line <1.0 mm, with bone proliferation and bridging callus (clinical healing)	6
Absence of fracture line	7
7. Resorption of the implant	
No evidence of resorption	1
Mild resorption	2
Full	3
8. Graft-host bone junction	
No connection	1
Cortex to trabecula	2
Cortex to cortex (one side)	3
Cortex to cortex (both sides)	4
9. Remodeling	
No remodeling	1
Remodeling of the intramedullary channel	2
Full	3
